# Effects of dredging on the vegetation in a small lowland river

**DOI:** 10.7717/peerj.6282

**Published:** 2019-01-22

**Authors:** Edyta Stępień, Andrzej Zawal, Paweł Buczyński, Edyta Buczyńska, Magdalena Szenejko

**Affiliations:** 1 Department of Plant Taxonomy and Phytogeography, Institute for Research on Biodiversity, Faculty of Biology, University of Szczecin, Szczecin, Poland; 2 Department of Invertebrate Zoology and Limnology, Institute for Research on Biodiversity, Faculty of Biology, University of Szczecin, Szczecin, Poland; 3 Department of Zoology, Maria Curie-Sklodowska University Lublin, Lublin, Poland; 4 Department of Zoology, Animal Ecology and Wildlife Management, University of Life Sciences in Lublin, Lublin, Poland; 5 Department of Ecology and Environmental Protection, Institute for Research on Biodiversity, Faculty of Biology, University of Szczecin, Szczecin, Poland

**Keywords:** Regulated river, Dredging, Management, Environmental change, Riparian vegetation

## Abstract

**Background:**

Conventional river engineering operations have a substantial influence on the fluvial ecosystem. Regulation and channelization generally reduce the physical heterogeneity of river beds and banks and the heterogeneity of habitats. They determine the character, diversity and species richness of plant communities. The effect of river regulation on vegetation has been repeatedly investigated, but few studies have been conducted within reaches of previously regulated rivers. The aim of this work is to expand and current knowledge about the impact of dredging on the vegetation of a regulated section of a lowland river.

**Materials & Methods:**

The study included pre-dredging (1 year before) and post-dredging surveys (results 1 and 2 years after dredging). The vegetation was analysed in terms of species composition, origin of species, life forms, distribution of Grime’s life strategies, and selected ecological factors. The Shannon–Wiener biodiversity index (H) and evenness were also analysed in each year of the study. The impact of dredging on the vascular flora was assessed by ‘before-after-control-impact’ (BACI) analysis.

**Results:**

The number of species and biodiversity as measured by the Shannon–Wiener index (H) increased in the analysed section of the river valley. However, enrichment of the flora was observed only on the floodplain, on the surface of the deposited dredging material, while the number of species in the river channel decreased, as dredging of the river bed and levelling of the banks had markedly reduced habitat diversity. Although species richness in the second year after the dredging approached the values recorded before the intervention, the absence of particularly species or phytocenoses associated with shallow river banks and sandbars was still observed. The change in habitat conditions and the destruction of the vegetation cover during the dredging enabled penetration by numerous previously unrecorded alien species of plants and apophytes. There was a perceptible increase in the role of therophytes in the flora. It is worth noting that the number of alien species and therophytes declined significantly in the second year after the dredging. Analysis of the proportions of species representing various life strategies showed that previously unrecorded species with the type R (ruderal) life strategy had appeared, representing by pioneer species occurring in frequently disturbed habitats. There was also a marked increase in the share of species representing the mixed C-R (competitive-ruderal) strategy, occurring in habitats with low levels of stress, whose competitive abilities are limited by repeated disturbances. By the second year after the dredging, however, these changes were largely no longer observed.

**Conclusions:**

Through appropriate maintenance of the regulated river, it can be rapidly recolonized by vegetation after the procedure, but it may lead to the loss of some species and phytocoenoses.

## Introduction

Rivers and river valleys have long been used in human economic activity. This has involved interference with their structure and function, in varying forms and intensity (Allan, 1998; Naiman et al., 2002). In spite of these transformations, they still constitute a key element of the natural environment and are among the most valuable ecological corridors and centres of biodiversity in a landscape transformed by man (Tockner et al., 1999; Nilsson & Svedmark, 2002). For this reason, research is conducted on the ecological state of rivers (Garófano-Gómez et al., 2011; Jusik et al., 2015; Płaska et al., 2016; Szoszkiewicz et al., 2017; Zawal et al., 2017; O’Briain, Shephard & Coghlan, 2018).

Conventional river engineering operations have a substantial influence on the fluvial ecosystem (Brookes, 1988). Regulation and channelization generally reduce the physical heterogeneity of river beds and banks (Ibisate, Ollero & Díaz, 2011) and change the natural flow regime (Naiman et al., 2002; Tena et al., 2017). They determine the character, diversity and species richness of plant communities (Baattrup-Pedersen et al., 2005; Myśliwy, 2010) and are responsible for changes in species composition and the introduction of alien species (Hood & Naiman, 2000; Myśliwy, 2014; Chen et al., 2017). Disturbed river habitats are considered to be more vulnerable to invasion of alien plants (Planty-Tabacchi et al., 1996; Lohmeyer & Sukopp, 2001). Human impact causes an increase in alien species (Richardson et al., 2007; Dyderski, Gdula & Jagodziński, 2015) and a decrease in native species in river ecosystems (Müller & Okuda, 1998). The literature data show a strong correlation between river regulation and management and decreases in species richness, the disappearance of stenoecic species, and the loss of biological diversity (Jongman, 1992; Nilsson & Berggren, 2000; Rambaud et al., 2009). This effect is linked to the change in the shape of the river bed and the decrease in the heterogeneity of habitats (Baattrup-Pedersen & Riis, 1999; Bondar-Nowakowska, Hachol & Lubczyński, 2013). On the other hand, some authors point out that the species richness and floristic composition of transformed and untransformed sections of river valleys are similar, with differences mainly involving area cover by certain species (Aguiar, Ferreira & Moreira, 2001; Caffrey, Monahan & Tierney, 2006). The effect of river regulation on vegetation has been repeatedly investigated (Merrit & Cooper, 2000; Sankey et al., 2015; Kui et al., 2017; Picco et al., 2017; Gill et al., 2018), but fewer works show the impact of interactions within previously transformed river sections (Henry & Amoros, 1996; Kaenel & Uehlinger, 1998; Baattrup-Pedersen, Larsen & Riis, 2002).

The aim of the study was to investigate the effect of one-time dredging of a regulated section of the lowland Krąpiel River (NW Poland) on vascular flora. The dredging was performed due to repeated extensive flooding onto neighbouring fields and meadows. The dredging involved deepening and levelling the bed and the banks of the river bed, shaping the river terrace and depositing the dredging material on its surface, as well as cutting down some of the trees and shrubs growing on the bank and in the river bed. The study was divided into two stages: pre-intervention (2008) and post-intervention (2009 and 2010). The following hypotheses were put forth: (1) dredging will result in species impoverishment of flora and a decrease in biodiversity; (2) it will allow alien species and native synanthropic species (apophytes) to penetrate the phytocenoses; and (3) it will cause a transformation of habitat conditions, which will alter the biological spectrum of the flora and favour species representing a ruderal strategy.

## Study Area

**Figure 1:**
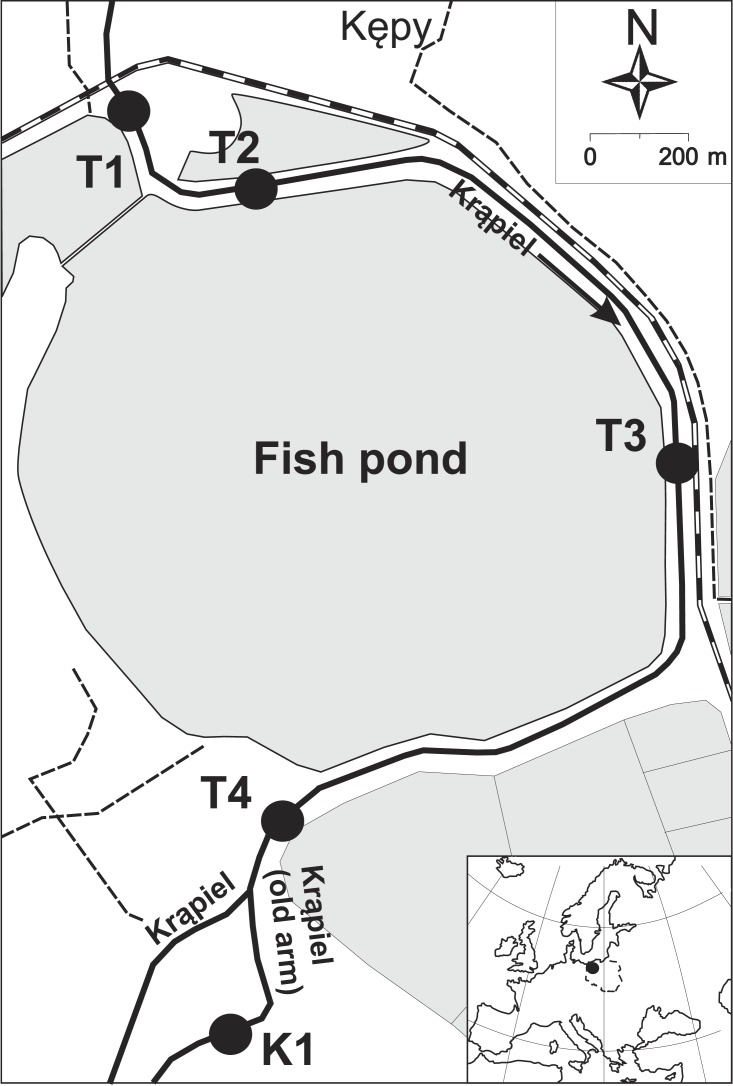
Map of the study area (Western Pomerania, Poland). T1-4, K1 –transects.

The Krąpiel River (length 65 km), located in north-western Poland, is the largest right tributary of the Ina River (Fig. 1). The Krąpiel River catchment, with an area of 596 km^2^, is classified as a small river (Bajkiewicz-Grabowska & Mikulski, 2017), and is mainly located in the Stargardzki District (West Pomerania Province). In the section from its sources to the Kania River confluence, it has the character of a lowland loess or loamy stream. From the Kania River to its mouth, it is classified as a lowland gravel river (Błachuta et al., 2010; European Parliament and the Council of the European Union, 2000). The river mostly flows through agricultural areas. A substantial section of it flows in a postglacial channel. From Pęzin, it transports water through a moraine plateau area in a deep gorge valley. Throughout nearly its entire length, the Krąpiel River valley is overgrown by deciduous forests.

Along the section analysed (from the railway bridge in Krzywnica to the fork in the river where it splits into two arms—the Krąpiel and the Stara Krapiel), the river flows through a complex of large fish ponds, adjacent to extensive areas overgrown with rush vegetation, regularly flooded in spring. Water inflow to the ponds is regulated by means of a dam located north of the village of Krzywnica. The river here has the character of a slow-flowing canal (current velocity from 0.002 to 0.16 m/s) with a depth of approximately 0.8 m and a mean width of 5 m. Before the dredging, the river had a muddy bottom, entirely overgrown by vegetation in places. The flood terraces, with a width of approximately 10 m and relatively steep slopes, were occupied by rush communities, and *Convolvuletalia sepium communities in the upper parts*. Bushes and tree stands dominated by willow developed locally over the water surface. The plant associations and communities that formed over the banks of the Krąpiel have been described by Stępień et al. (2015). Fish ponds situated on both sides of the river were created in the 1970s. This reach of the river was deepened and regulated at that time. After this, shrubs and young trees that significantly inhibited the water flow were cut down sporadically, every few years. Along the right river terrace an embankment was formed, on top of which there is a road used for the purposes of fish farming. Due to repeated extensive flooding onto neighbouring fields and meadows, the need arose to increase the magnitude of river flow of this reach of the river. Dredging of the river bed combined with clearing of trees and bushes occupying its banks was performed in February 2008. Only trees and bushes reducing the water flow of the river were cut down. Prior to the dredging, the trees and bushes did not form a dense canopy over the river channel and did not cut off access to light, so that lush vegetation developed in the river bed. During the dredging, the river bed and the surface of the floodplain were reshaped. The resulting dredging material was mostly deposited on the right bank of the river in an evenly distributed layer. Owing to the deepening of the river bed and the elevation and levelling of the terrace, there were no spring floods within the floodplain of the river during the two consecutive years of research.

## Material and Methods

### Field survey

The field research was conducted over three seasons: in July 2008, 2009 and 2010. The dredging we examined the results of was done in February 2008. A pre-dredging survey was conducted during the first year of the research. Over the following two years, changes in the vegetation after the dredging were observed. The research concerned the section of the Krąpiel River below the dam in Krzywnica, from the former railway bridge to the fork of the Krąpiel and Stara Krąpiel Rivers (N: 53.422909°; E: 15.193214° to N: 53.408067°; E: 15.198897°) (Fig. 1).

Five transects of 25 m were selected on this section of the river, covering the river channel together with the floodplain to the top of the embankment. One transect ran through undredged locations (control transect) K1, and the remaining transects at dredged sites: T1-T4 (Fig. 1). The control transect was selected on the section of the river with the most similar vegetation structure to the study area. The control transect has not been influenced by dredging, although it is located downstream of the dredged part of the river. Phytosociological relevés were made in each transect, covering all of the plant communities formed within them. The number of relevés in the transect depended on the heterogeneity of the phytocoenoses, but was not less than five. The relevés were made according to Braun-Blanquet (1964) with additional categories for cover-abundance scales (Barkman, Doing & Segal, 1964): r (one or a few individuals), + (occasional), 1 (abundant and less than 5%), 2m (very abundant and cover about 5%), 2a (5–12.5%), 2b (12.5–25%), 3 (25–50%), 4 (50–75%), and 5 (75–100%). A database was created using Turboveg software for Windows (Hennekens & Schaminee, 2001).

### Statistical analysis

Data related to species abundance were used to compute the Shannon–Wiener diversity index (H) (Shannon & Weaver, 1949). For data analysis, cover-abundance values were transformed into the 1-9 ordinal scale of van der Maarel (1979) as follows: ‘r’–1, ‘+’–2, ‘1’–3, ‘2m’–4, ‘2a’–5, ‘2b’–6, ‘3’–7, ‘4’–8, and ‘5’–9. Statistical significance of differences was determined by a test comparing values for the Shannon–Wiener index (Zar, 1984) developed by Jayaraman (1999). The Shannon–Wiener diversity index (H) was used to calculate species evenness (Pielou, 1966), to determine the uniformity of the distribution of species within the habitats.

The significance of the differences in the number of species between the three years was calculated using the Mann–Whitney *U*-test using Statistica 12 PL software (Statsoft Polska, 2014).

The impact of dredging on the vascular flora was assessed by ‘before-after-control-impact’ (BACI) analysis, which compares data obtained in the control transect (K1) with data obtained in the impacted transects (T1-T4) before and after the intervention (2008 and 2009, 2009 and 2010, 2008 and 2010). BACI tests for a BA x CI interaction (Smith, Orvos & Cairns. Jr, 1993). The impact of dredging was analysed by testing the abundance of vascular plants, where each species was recorded and expressed as the cover of the species at a given site. The cover of each species was used as the dependent variable. BACI was tested using a generalized mixed model (GLMM) with a log link and a negative binomial distribution. This should be used when dependent variables show high variation. We used species as a random effect. R software was used for the statistical computations (R Development Core Team, 2014), with the lme4 package (Bates et al., 2014). Probability lower than 0.05 was used as the significance threshold.

### Origin of species

Species were classified into native—those that have appeared or are present in a given area as a result of natural processes, i.e., without human intervention (spontaneophytes, found solely in plant communities independent of human interference, and apophytes, found in plant communities existing owing to constant or periodical human intervention) and alien—living outside their native distributional range, having arrived through human activity (archaeophytes, introduced before ca. 1500 and neophytes, introduced after ca. 1500) (Pyšek, Sádlo & Mandák, 2002). Neophytes were also classified according to their invasion status, as casual or naturalized (Pyšek et al., 2004). Status of alien plants was given with respect to Western Pomerania as the area of reference, using data provided by Zając & Zając (2001) and Mirek et al. (2002).

### Estimation of indices

To evaluate the changes in habitat conditions resulting from the dredging, the species recorded were analysed in terms of indicator values Ellenberg et al. (1992) adapted by Zarzycki et al. (2002) for Polish climatic and edaphic conditions). Five indices were taken into account: light value L (5-grade scale), soil moisture value W (6-grade scale), trophy value Tr (5-grade scale), soil (water) acidity (pH) R (5-grade scale) and soil granulometric value D (5-grade scale). The species were grouped according to the year of the study (2008–2010) and their location in the river valley (river channel or terrace). The data obtained from the four transects were analysed together. As suggested by the creators of the scale (Roo-Zielińska, 2012), for species described by more than one numerical value for individual indices, the mean value for the range of indicator values was adopted. Since the data used in the analysis were not transformed and did not have normal distribution, it was not possible to use a parametric test, so statistical significance between groups was tested by the nonparametric Kruskal–Wallis test using Statistica 12 PL software (Statsoft Polska, 2014).

**Figure 2:**
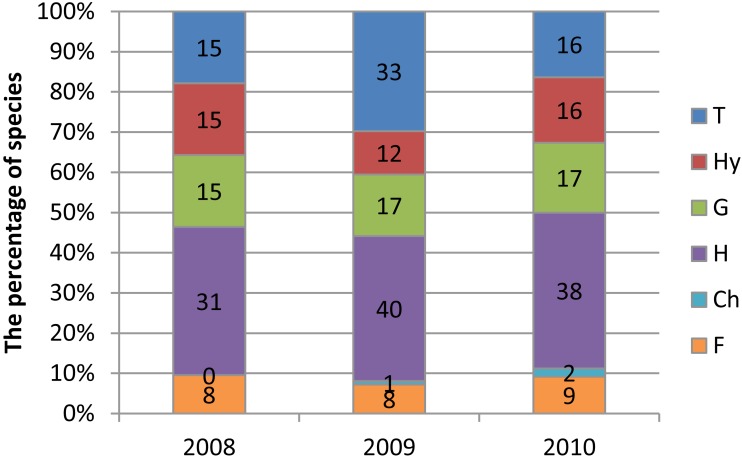
Frequency of species representing Raunkiaer’s life forms in transects T1-T4 (Krąpiel River) in the years 2008–2010. T, therophytes; Hy, hydrophytes and helophytes; G, geophytes; H, hemicryptophytes; Ch, chamaephytes; F, phanerophytes.

Grime’s method (2001) was used to determine whether the dredging had caused a change in the proportions of species representing particular live strategies during the three years of the study. According to Grime, the life strategy of plants is determined by three kinds of selection pressure—C (competitive), for species resistant to interspecific competition, R (ruderal), for pioneer species occurring on disturbed habitats, and S (stress-tolerant), for plants resistant to stress factors, and by several secondary strategies (CS–competitive stress-tolerant; CR–competitive ruderal; SR–stress-tolerant ruderal; and CSR–competitive stress-tolerant ruderal). The biological spectrum of flora is a sensitive indicator of environmental changes (Jackowiak, 1998). To ascertain these changes, the percentages of Raunkiaer’s life forms (explanations presented in the caption to the Fig. 2) in each year of the study were calculated. The data obtained from the four transects were analysed together.

### Nomenclature of plant species and plant communities

Species nomenclature was adopted following Mirek et al. (2002). The classification of plant communities was taken from Brzeg & Wojterska (2001).

## Results

### Number and origin of species

A total of 137 vascular plant species were recorded within the four transects in the years 2008–2010, including 113 native taxa (41 spontaneophytes and 72 apophytes) and 24 alien species (16 archaeophytes, four naturalized neophytes and four casual neophytes).

**Figure 3:**
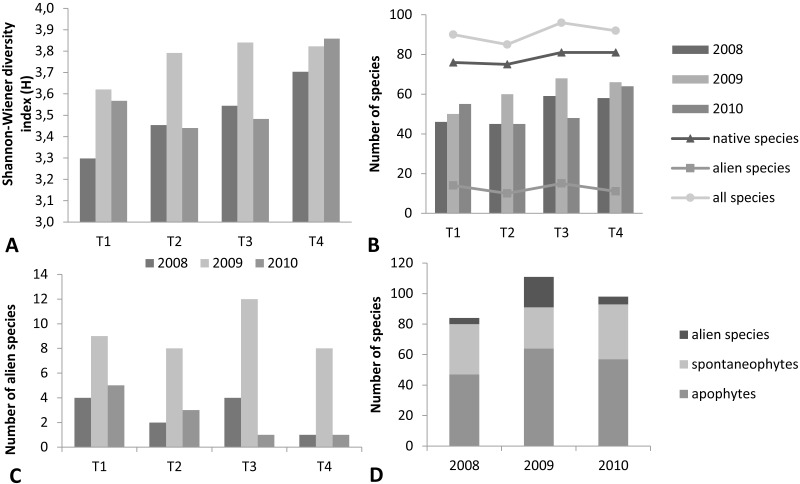
Number of vascular plant species and biodiversity in individual transects (Krąpiel River) in the years 2008–2010. (A) Shannon–Wiener diversity index (H), (B) number of species in transects in each year (bars) and number of species in transects in the three years together (lines), (C) number of alien species, (D) number of alien and native species (T1-4 numbers of the transects).

**Figure 4:**
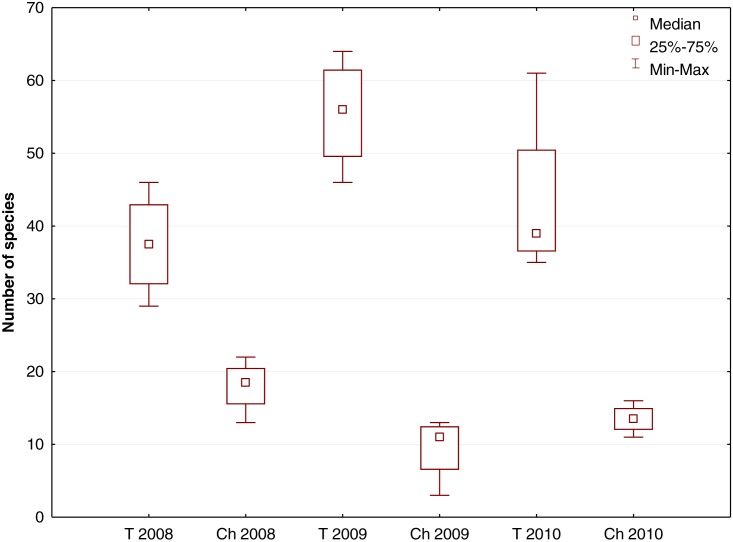
Box-and-whisker plot showing the number of species growing in the river channel (Ch) and flood terrace (T) in transects T1–T4 (Krąpiel River) in the years 2008–2010.

The total number of species increased after the dredging, particularly in the first year (Fig. 3B, Fig. 3D). However, the separate analyses of the river channel and the floodplain yielded different results. The number of species in the river channel itself decreased (Fig. 4). The increase of the number of species in the studied section of the river resulted from the increase of species abundance of terrace vegetation. The differences in the number of species were statistically significant only between 2008 and 2009, within both the channel (*p* = 0.04, *Z* = 2.0329) and the terrace (*p* = 0.042, Z = -2.0329). In the control transect 99 species (river channel –18, river terrace –89) in 2008, 90 species (river channel –19, river terrace –79) in 2009 and 98 species (river channel –21, river terrace –88) in 2010 were noted.

**Figure 5:**
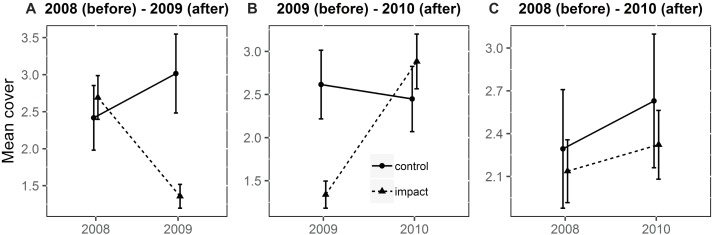
Mean cover of vascular plants within the river channel (back-transformed) in the BACI design obtained from the fitted model. (A) 2008 (before)-2009 (after); (B) 2009 (before)-2010 (after) (C) 2008 (before)-2010 (after). The interaction is significant between 2008 and 2009 and between 2009 and 2010 (*p* < 0.05); the interaction is not significant between 2008 and 2010 (*p* > 0.05).

**Figure 6:**
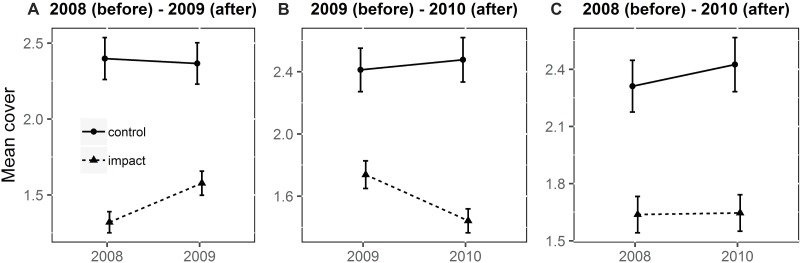
Mean cover of vascular plants on the river terrace (back-transformed) in the BACI design obtained from the fitted model. (A) 2008 (before)-2009 (after); (B) 2009 (before)-2010 (after) (C) 2008 (before)-2010 (after). The interaction is significant between 2008 and 2009 and between 2009 and 2010 (*p* < 0.05); the interaction is not significant between 2008 and 2010 (*p* > 0.05).

The BACI interaction showed that the cover of vascular plants changed after the dredging. BACI analysis showed a significant effect of the dredging within the river channel between 2008 and 2009 (Table 1). After the intervention, the mean cover at the impact site decreased (Fig. 5). A significant effect was observed between 2009 and 2010. After the experiment, the mean cover at the impact site increased. The BACI interaction showed no effect of the dredging between 2008 and 2010. On the river terrace, the BACI interaction showed that the dredging had a significant effect between 2008 and 2009 (Table 2). After the dredging, the mean cover at the impact site increased while cover at the control site remained stable (Fig. 6). The BACI showed a significant effect of the dredging between 2009 and 2010. After the intervention, the mean cover at the impact site decreased while cover at the control site remained stable. The BACI interaction showed no effect of the intervention between 2008 and 2010. The dredged site had significantly lower cover.

**Table 1:** BACI analysis of the impact of dredging on vascular flora within the river channel. Parameter estimation in the fitted mixed (negative binomial error distribution) model where cover was the dependent variable.

	Estimate	Std. error	*z*	*P*
**2008–2009**				
(Intercept)	0.882	0.181	4.890	**<0.001**
BA(2009)	0.221	0.220	1.004	0.315
CI(impact)	0.108	0.176	0.612	0.541
BA(2009):CI(impact)	−0.906	0.243	−3.723	**<0.001**
**2009–2010**				
(Intercept)	0.961	0.152		
BA(2010)	−0.066	0.178	−0.372	0.710
CI(impact)	−0.669	0.151	−4.446	**<0.001**
BA(2010):CI(impact)	0.833	0.198	4.212	**<0.001**
**2008–2010**				
(Intercept)	0.830	0.181		
BA(2010)	0.137	0.230	0.595	0.552
CI(Iimpact)	−0.071	0.179	−0.394	0.693
BA(2010):CI(impact)	−0.054	0.248	−0.217	0.828

**Table 2:** BACI analysis of the impact of dredging on vascular flora on the river terrace. Parameter estimation in the fitted mixed (negative binomial error distribution) model where cover was the dependent variable.

	Estimate	Std. error	*z*	*P*
**2008–2009**				
(Intercept)	0.875	0.058		
BA(2009)	−0.014	0.063	−0.214	0.831
CI(impact)	−0.596	0.063	−9.420	**<0.001**
BA(2009):CI(impact)	0.191	0.083	2.308	**0.021**
**2009–2010**				
(Intercept)	0.880	0.058		
BA(2010)	0.027	0.064	0.414	0.679
CI(impact)	−0.327	0.064	−5.107	**<0.001**
BA(2010):CI(impact)	−0.214	0.085	−2.526	**0.012**
**2008–2010**				
(Intercept)	0.838	0.059		
BA(2010)	0.048	0.066	0.727	0.467
CI(Iimpact)	−0.344	0.067	−5.141	**<0.001**
BA(2010):CI(impact)	−0.043	0.088	−0.483	0.629

The number of alien species increased considerably in the first year after the dredging (Fig. 3C). In the transformed habitat 13 archaeophytes were recorded (*Anchusa officinalis*, *Apera spica-venti*, *Bromus sterilis*, *Capsella bursa-pastoris*, *Echinochloa crus-galli*, *Erysimum cheiranthoides*, *Fallopia convolvulus*, *Lactuca seriola*, *Matricaria maritima* subsp. *inodora*, *Melandrium album*, *Papaver dubium*, *Sonchus oleraceus* and *Vicia hirsuta*), four casual neophytes (*Avena sativa*, *Brassica napus* subsp. *napus*, *Phacelia tanacetifolia* and *Triticum eastivum*) and three naturalized neophytes (*Bidens frondosa*, *Echinocystis lobata* and *Elodea canadensis*). A pronounced decrease in the number of alien species was recorded in the second year after the dredging. No casual neophytes were observed, while *Elodea canadensis* was noted in the water and 4 archaeophytes were found on the river terrace (*Ballota nigra*, *Berteroa incana*, *Melandrium album* and *Vicia hirsuta*).

The number of native species also increased after the dredging, from 80 to 91 (Fig. 3D). Among these, however, the abundance of apophytes increased (e.g., *Echium vulgare*, *Erodium cicutarium*, *Rumex obtusifolius* and *Stellaria media*), whereas the number of spontaneophytes decreased (e.g., *Butomus umbellatus*, *Callitriche cophocarpa*, *Glyceria maxima* and *Rumex hydrolapathum*).

### Biodiversity indices

The values for the Shannon–Wiener diversity index (H) (Fig. 3A) and the evenness index (Table 3) in individual transects changed over the three years of the study. The differences in the H index were statistically significant between 2008 and 2009 and between 2008 and 2010 within the first transect (*p* < 0.01), and between 2008 and 2009 (*p* < 0.01) and 2009 and 2010 (*p* < 0.01) within the second transect. In the third transect, statistically significant differences were noted between 2008 and 2009 (*p* < 0.01) and between 2009 and 2010 (*p* < 0.01). The differences in the values for the Shannon–Wiener index in the fourth profile were statistically significant between 2008 and 2010 (*p* < 0.01) and between 2008 and 2009 (*p* = 0.05).

**Table 3:** Species evenness calculated on the basis of the Shannon–Wiener diversity index (H) in individual transects (Krapiel River) in the years 2008–2010. T1-4 numbers of the transects.

	Transect 1	Transect 2	Transect 3	Transect 4
2008	0,86	0,91	0,87	0,91
2009	0,93	0,93	0,91	0,91
2010	0,89	0,90	0,90	0,93

### Ecological indices and life strategy

Analysis of the proportions of life forms revealed a pronounced increase in the proportion of therophytes and a decrease in that of hydrophytes in the first year after the dredging (Fig. 2).

**Figure 7:**
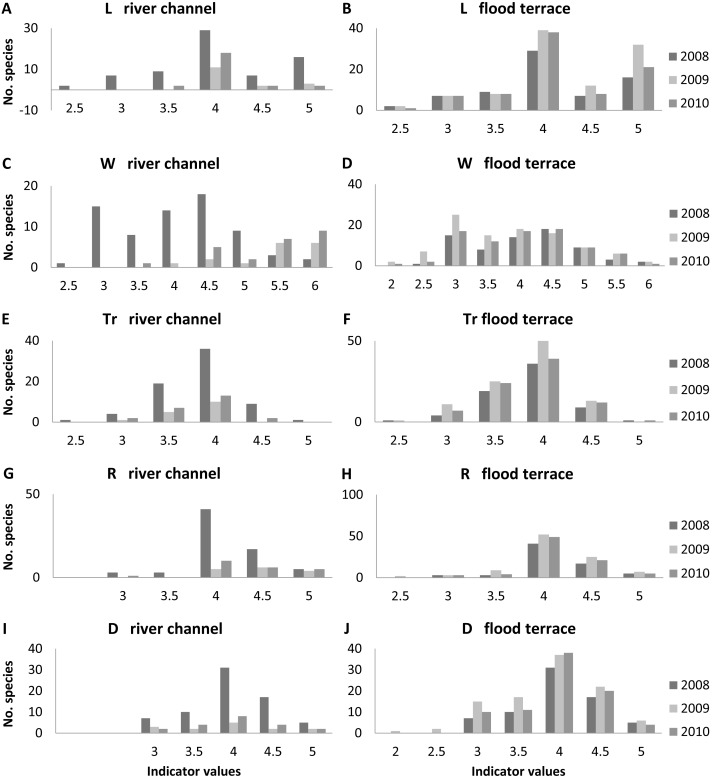
Distribution of indicator values for species growing in the river channel and flood terrace in each year of research in transects T1-T4. (A) L river channel; (B) L flood terrace; (C) W river channel; (D) W flood terrace; (E) Tr river channel; (F) Tr flood terrace; (G) R river channel; (H) R flood terrace; (I) D river channel; (J) D flood terrace. L, light; W, moisture; Tr, trophy; R, soil (water) acidity (pH); D, soil granulometric value.

The ranges of values for the ecological indices of the species noted in the river valley are wide, but the largest number of species represents a narrow range of individual parameters (Fig. 7).

On the river terrace the greatest number of species represent habitats with moderate and full light, mesic and moist in terms of soil moisture, with neutral pH, trophically rich (eutrophic), and in terms of the granulometric index, characterized by sandy loam and silt deposits. The differences between years were statistically insignificant.

Within the river channel the greatest number of species represent habitats with moderate and full light, trophically rich, and in terms of the granulometric index, characterized by sandy loam and silt deposits. For these indices (L, light, Tr, trophy and D, soil granulometric value), the differences between the three years of the study were statistically insignificant. The soil moisture index (W) for the recorded species indicates a change in habitat. Species characteristic of mesic, moist and wet habitats were dominant in 2008 (such as *Agrostis gigantea* and *Erysimum cheiranthoides*), and species typical of wet and aquatic habitats in 2009 and 2010 (such as *Rorippa amphibia* and *Sparganium emersum*). The differences obtained were statistically significant (Kruskal–Wallis test: H (2, *N* = 110) = 44.65 *p* < 0.01). However, the statistical significance was due to the differences between the years 2008 and 2009 (*p* < 0.01) and between 2008 and 2010 (*p* < 0.01), while the differences between 2009 and 2010 were statistically insignificant. The values for the pH index (R) changed as well; neutral habitat species were dominant in 2008, but both neutral and alkaline habitat species dominated in the following years. The differences were statistically significant (Kruskal–Wallis test: H (2, *N* = 107) = 9.34 *p* = 0.01); however, the statistical significance was due to the differences between the years 2008 and 2009 (*p* = 0.01), while the differences between the remaining years were statistically insignificant.

**Figure 8:**
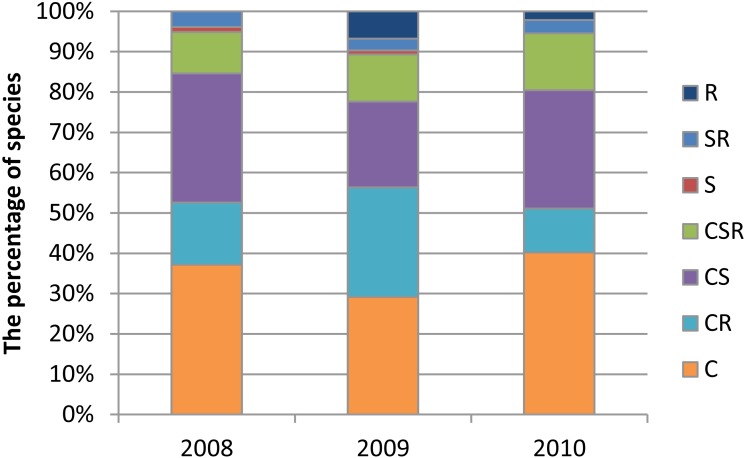
The percentage of species representing Grime’s strategy types in vegetation growing in the Krąpiel valley in the years 2008–2010 in transects T1-T4. C, competitive; S, stress tolerant; R, ruderal.

The intervention caused a change in the proportions of species representing different life strategies (Grime, 2001). In the first year after the dredging, there was a notable increase in the percentage of species representing the mixed C-R strategy, and previously unrecorded species with life strategy R appeared—pioneer species, which occur in frequently disturbed habitats (Fig. 8).

## Discussion

The investigated section of river, which was regulated in the 1970s, despite being managed in accordance with the needs of the neighbouring fish pond, has retained many of its natural qualities. The relatively diverse bottom and banks, with alluvia and sections with a faster and slower current, enabled the formation of varied phytocoenoses. The dredging, which involved deepening the river bed and shaping its bottom and banks and the surface of the floodplain, caused changes in the structure and character of the vegetation.

### Species richness and biodiversity indices

The study carried out in the Krąpiel River valley (in the river channel and on the river terrace together) showed an increase in the number of species and in the Shannon–Wiener index (H) and evenness index after the technical procedures. These results seem to diverge from research conducted in other areas. Many studies indicate a negative impact of regulation and dredging on species richness and biodiversity (Baattrup-Pedersen & Riis, 1999; Sand-Jensen et al., 2000; Pedersen, Baattrup-Pedersen & Madsen, 2006; Omelchuk & Prots, 2015; Bączyk et al., 2018). Some analyses show no differences in the number of species between natural and regulated reaches of river, but only changes in the dominance of certain species (Aguiar, Ferreira & Moreira, 2001). However, it should be emphasized that the present work deals with changes within a previously regulated part of a river. Caffrey, Monahan & Tierney (2006), in their study on the impact of management practices on regulated watercourses, did not find that they significantly affected the overall structure and richness of plant communities.

However, river valleys cannot be considered only as a whole. The vegetation in the river bed and on the surface of the floodplain responded differently to the technical procedures. In the first year after the dredging, a marked decrease in species richness was observed, but by the following year it had increased. BACI analysis showed that the effect of the dredging on the number of species observed between 2008 and 2010 was statistically non-significant. However, it is important to note not only the number of species, but species composition as well. The deepening of the river channel and straightening of the banks notably reduced habitat diversity, thereby impoverishing the plant communities and the species composing them. Especially species and phytocenoses associated with shallow river banks and sandbars. The species *Callitriche cophocarpa*, *Fontinalis antipyretica*, *Butomus umbellatus*, *Acorus calamus*, *Glyceria fluitans*, *G. maxima*, *Rumex hydrolapathum*, *Stachys palustris*, *Veronica beccabunga* and *Rumex hydrolapathum* were not recorded in the river channel after the dredging. A decrease in the number of species in the river bed have also been observed by other authors (Rambaud et al., 2009; Steffen et al., 2013) and were associated with a decrease in the heterogeneity of transformed river beds. Wade (1993) noted the uniformity of vegetation along a dredged channel. Pedersen, Baattrup-Pedersen & Madsen (2006) observed that the preservation of shallow and wide banks contributes to greater species richness. Bondar-Nowakowska, Hachol & Lubczyński (2013) also observed that the value of the biodiversity index and the number of macrophytic species in the river bed were associated with the degree of meandering and the shape of the cross section of the river bed. In addition, the dredging process is associated with a change in the area cover of species. Periodic removal of macrophytes from the river bed results in dominance of the habitat by species resistant to transformations (Kaenel & Uehlinger, 1999). These species include *Sparganium emersum*, which was dominant in regulated Danish watercourses (Baattrup-Pedersen & Riis, 1999). Likewise, in the Krąpiel River it quickly dominated the river bottom (together with *Sagittaria sagittifolia*) that had been cleared of vegetation.

A different process was observed on the surface of the floodplain. BACI analysis showed no statistically significant difference in the number of species before the dredging and in the second year after the intervention (2008–2010). Before the dredging, communities of rushes covered a large area, with dominant species with a high degree of coverage. These species belong to species-poor communities. After the dredging, the vegetation within the floodplain was mainly represented by riparian therophytes (the association Chenopodietum rubri), with *Polygonum lapathifolium* subsp. *lapathifolium* as the dominant species. Communities of the Bidentetea tripartite class are floristically rich (Kiesslich, Dengler & Berg, 2003). The deposition of dredging material allowed new species to penetrate to the surface of the terrace and caused an increase in the number of species as compared to before the disturbance. The increase in the uniformity of area cover by species can also be seen in the increase in evenness. In northern Sweden, on the other hand, a decrease in species richness was noted as a result of dredging (Malm-Renöfält & Nilsson, 2008).

### Origin of species

River valleys are floristically rich due to their specificity and habitat diversity (Müller, 1995a). Disturbances caused by floods and the availability of nutrient resources facilitate penetration by alien species (Pyšek & Prach, 1993; Stohlgren et al., 1998). Furthermore, the number of alien species increases in areas transformed by human activity (Nilsson & Berggren, 2000; Tabacchi & Planty-Tabacchi, 2003; Pyšek et al., 2010).

The destruction of the vegetation cover and deposition of dredging material on the surface of the terrace permitted the invasion of alien species. The alien species mainly penetrated the summer therophyte communities (Bidentetea tripartitae class) dominating the surface of the terrace. These communities, particularly in disturbed areas, are susceptible to invasion by alien species (Lohmeyer & Sukopp, 2001). The development of new habitats and their transformation as a result of river regulation favour the appearance of alien species (Bond & Lake, 2003; Barbosa et al., 2006; Richardson et al., 2007). This process mainly affects the surface of the terrace, as observed by Aguiar, Ferreira & Moreira (2001) In more stable plant communities they would have no chance to develop (Brzeg & Ratyńska, 1983). The dominant group among them is archaeophytes, which have the ability to penetrate from neighbouring farmland, such as *Anchusa officinalis*, *Apera spica-venti*, *Bromus sterilis* and *Papaver dubium*. Penetration of alien species from human settlements and agricultural areas within river valleys has been observed many times (Müller, 1995a; Müller & Okuda, 1998; Tokarska-Guzik, 2003; Myśliwy, 2008; Stępień, 2009). These farmlands are also the source of casual neophyte diaspores (*Avena sativa*, *Brassica napus* subsp. *napu* s, *Phacelia tanacetifolia* and *Triticum aestivum*), whereas the naturalized neophytes noted belonged to species that spread along river valleys (Tokarska-Guzik, 2005). Two new species were recorded after the dredging—*Bidens frondosa* and *Echinocystis lobata*. Riverside areas newly colonized by vegetation are richer in terms of the number of alien species than longer-lasting vegetation patches (Planty-Tabacchi et al., 1996).

In the second year after the dredging there was a substantial decrease in the number of species of alien origin, and a similar sharp decrease in the surface area of patches of summer therophytes. These phytocoenoses were mainly replaced by rush communities and, in places, riparian tall herb fringe communities. Alien pioneer species which appeared in the sections of the river valley that were cleared of vegetation gave way the following year to species of higher successional stages. The dense vegetation cover prevented the growth of photophilic pioneer species (Planty-Tabacchi et al., 1996; Stromberg, Gengarelly & Rogers, 1997).

It should be emphasized, however, that the number of alien species was not high either before or after the dredging. This is due to the nature of the river. Small rivers have fewer alien species than large rivers (Stępień, 2009; Stępień, 2010), where even more than 50 alien species were recorded (Hood & Naiman, 2000; Dyderski, Gdula & Jagodziński, 2015). Moreover, the Krąpiel runs mainly through agricultural and forest areas, with meadows and forests covering a large area of the valley. The share of species of alien origin in the flora is most often strongly correlated with the degree of ecosystem transformation (Krawczyk, 2011). Kenophytes in particular are most abundant in urban areas (Sudnik-Wójcikowska, 1998), which may be a source of them for sections of river valleys below them. In addition, a positive correlation has been shown between species richness and the number of alien species (Krawczyk, 2011; Dyderski, Gdula & Jagodziński, 2015).

It should also be emphasized that after the dredging the flora was enriched not only by alien species but also by apophytes. Even in the first year of the study these species formed the most numerous group, a tendency which was intensified following the dredging. Native species with a wide ecological amplitude are characterized by a significant ability to penetrate transformed habitats, which constitute surrogate habitats for them (Zając & Zając, 2009) and are an important component of the flora of various areas (Sukopp, 2008).

### Ecological indices and life strategy

The works carried out in the river valley caused a change in habitat conditions, as indicated by the analysis of the proportion of indicator species (Ellenberg et al., 1992). Interestingly, significant differences were observed within the river bed for the moisture (W) and pH (R) indices. This was a direct consequence of the impoverishment of the habitat structure in the river bed, particularly at its edges. Differences in moisture after a disturbance have been noted in the Bzura river valley (Kopeć et al., 2014). Kryszak, Grynia & Kryszak (2004), in transformed sections of the Warta River valley within the floodplain, found an increase in the proportion of mesic habitat species. This process was not observed in the Krąpiel valley, but it cannot be ruled out after a longer time period.

Müller (1995a), in an amphibious zone significantly decreased by human impact, observed changes in ecological conditions. In addition, a decrease in river dynamics results in an increase in species common to moist and ruderal sites. In the section of the river Krąpiel investigated in this study, comparison of the proportions of species in terms of the life strategies they represent (Grime, 2001) indicates changes in the level of environmental disturbances (Roo-Zielińska, Solon & Degórski, 2007). The drastic interference with the vegetation and habitat structure led to the appearance of species representing the type R life strategy (the ruderal type), such as *Capsella bursa-pastoris*, *Phacelia tanacetifolia*, *Polygonum avivulare* and *Vicia hirsuta*, as well as a pronounced increase in the number of species with the mixed C-R strategy (characterized by resistance to competition limited by repeated disturbances), such as *Apera spica-venti*, *Bidens frondosa*, *Bromus sterilis*, *Galeopsis bifida*, *Papaver dubium*, *Sonchus asper* and *Stellaria media*. The vast majority of these species were apophytes and archaeophytes, which was also observed by Müller (1995a; 1995b). Analysis of the proportions of species representing different life forms shows that the flora of the valley was mainly enriched by therophytes, but short-lived perennials were also present among the species representing the R and C-R types of life strategy. As disturbances intensify, species with a ruderal strategy play a greater role, reducing the share of dominant species and thereby increasing species diversity (Pierce et al., 2007). In the following year, when there was no longer any work being conducted in the river valley, there was a marked decrease in the proportion of species representing the R and C-R strategy types. The factor of interspecies competition came into play on the surfaces where the plant cover was restored (Symonides, 1974; Müller & Okuda, 1998). The pioneer therophyte communities gave way to communities of subsequent stages of succession.

## Conclusions

The number of species and biodiversity as measured by the Shannon–Wiener index (H) increased in this section of the river valley as a result of dredging. However, enrichment of the flora was observed only within the floodplain on the surface of the deposited dredging material, while in the river bed the number of species decreased—the deepening of the bottom and levelling of the banks had significantly reduced habitat diversity. Although in the second year after the dredging species richness approached the values from before the intervention, particularly species or phytocenoses associated with shallow river banks and sandbars were still absent.

The fertile surface of the floodplain, uncovered by vegetation and without natural flooding, enabled the penetration of previously unrecorded species. The number of alien species increased significantly, dominated by archaeophytes, diaspores of which came from neighbouring agricultural areas. The number of native species—apophytes—increased as well, while the number of spontaneophytes decreased. It is worth noting that the number of alien species declined significantly in the second year after the dredging.

The dredging caused a change in the proportions of species representing different life strategies (Grime, 2001). An increase in the share of species representing the mixed C-R strategy was observed, and the appearance of previously unrecorded species with the R life strategy—pioneer species, which occur in habitats that are frequently disturbed. This resulted in a change in the biological spectrum of the flora—an increase in the number of therophytes and a decrease in the proportion of hydrophytes, but by the second year after the intervention the number of therophytes had decreased and the number of hydrophytes had increased. The intervention caused a change in habitat parameters within the river bed (the soil moisture (W) and pH (R) indicators).

Appropriate planning and execution of procedures to prevent flooding of a small, lowland river that has previously undergone regulation may reduce losses in species richness and vegetation diversity. If procedures are carried out rarely and on short sections of the watercourse, plants that were present prior to the procedure will be able to recolonize it, but some elements of the flora may be lost.

##  Supplemental Information

10.7717/peerj.6282/supp-1 Supplemental Information 1 List of identified species of vascular plants in particular transects: T1-T4, K1

## Additional Information and Declarations

### Data Availability

The following information was supplied regarding data availability: The raw data is available as a Supplemental File.
